# Digital twin-driven smart supply chain

**DOI:** 10.1007/s42524-021-0186-9

**Published:** 2022-01-27

**Authors:** Lu Wang, Tianhu Deng, Zuo-Jun Max Shen, Hao Hu, Yongzhi Qi

**Affiliations:** 1 Department of Industrial Engineering, Tsinghua University, Beijing, 100084 China; 2 Faculty of Engineering and Faculty of Business and Economics, University of Hong Kong, Hong Kong, 999077 China; 3 Department of Industrial Engineering and Operations Research and Department of Civil and Environmental Engineering, University of California, Berkeley, Berkeley, CA 94720 USA; 4 JD.COM, Beijing, 100101 China

**Keywords:** digital twin, supply chain management

## Abstract

Today’s supply chain is becoming complex and fragile. Hence, supply chain managers need to create and unlock the value of the smart supply chain. A smart supply chain requires connectivity, visibility, and agility, and it needs be integrated and intelligent. The digital twin (DT) concept satisfies these requirements. Therefore, we propose creating a DT-driven supply chain (DTSC) as an innovative and integrated solution for the smart supply chain. We provide background information to explain the DT concept and to demonstrate the method for building a DTSC by using the DT concept. We discuss three research opportunities in building a DTSC, including supply chain modeling, real-time supply chain optimization, and data usage in supply chain collaboration. Finally, we highlight a motivating case from JD.COM, China’s largest retailer by revenue, in applying the DTSC platform to address supply chain network reconfiguration challenges during the COVID-19 pandemic.

## 1 Introduction

Today's supply chain is becoming complex and fragile. Hence, supply chain managers need to create and unlock the value of the smart supply chain. For example, the 2020 Coronavirus outbreak threatened the supply chain domestically and globally and significantly affected the supply chain, including supply shortages, sourcing limitations, logistical delays, and demand reductions. Automakers, such as General Motors, Nissan Motor, and Fiat Chrysler, halted production in other countries due to the supply shortage caused by China's manufacturing shutdown (Wayland, 2020). Apple has also reported that it does not expect to achieve its second quarter in 2020 forecasts for revenue (Lucas, 2020). Given that most iPhones are assembled in China, the Coronavirus outbreak has led to Apple's global constraints on the supply side. On the demand side, closed Apple stores have resulted in lower demand in China.

To protect the supply chain from this type of disruption, firms must continuously monitor the outbreak, check inventory and logistical hubs, and respond rapidly to changing circumstances. In addition, to evaluate the overall risk and impact, firms must assess upstream suppliers and downstream customers. In the long term, firms should rethink their supply chain structure and strategy and conduct simulation exercises to prevent future incidents (Hippold, 2020). The challenges faced by supply chain managers in the pandemic demonstrate the requirements of the smart supply chain:

- **Connectivity.** Connectivity is the ability to connect all enterprises, products, properties, and other valuable items in the supply chain in order to provide comprehensive information and to monitor the marketing status, intra-enterprise operations, and inter-enterprise communications (Butner, 2010; Wu et al., 2016).
- **Visibility.** Visibility is the ability to keep track of the flow of materials, finances, and information throughout the supply chain (Butner, 2010; Busse et al., 2021). Supply chain managers must have access to real-time data related to production, inventory, logistics, and marketing in order to identify where and how products are stocked and when and how products are sold to consumers.
- **Agility.** Agility is the quick ability to detect changes, collect relevant data, analyze opportunities and threats, make optimal decisions, implement these decisions,

Lu WANG, Tianhu DENG (✉)
Department of Industrial Engineering, Tsinghua University, Beijing 100084, China
E-mail: deng13@mail.tsinghua.edu.cn

Zuo-Jun Max SHEN
Faculty of Engineering and Faculty of Business and Economics, University of Hong Kong, Hong Kong 999077, China; Department of Industrial Engineering and Operations Research and Department of Civil and Environmental Engineering, University of California, Berkeley, Berkeley, CA 94720, USA

Hao HU, Yongzhi QI
JD.COM, Beijing 100101, China

The authors are grateful for the financial support from the National Key R&D Program of China (Grant No. 2018YFB1700600).

and modify operations accordingly (Gligor et al., 2019; Seyedghorban et al., 2020).

• **Integrated.** An integrated supply chain shares information and makes decisions jointly across different stages of the supply chain (Wu et al., 2016).

• **Intelligent.** An intelligent supply chain makes large-scale, optimal decisions and uses predictive analytics to protect the supply chain from future risks (Butner, 2010; Wu et al., 2016; Busse et al., 2021).

Many organizations desire to unlock the value of the smart supply chain but do not know how to begin. Creating a digital twin-driven supply chain (DTSC) is an innovative and integrated solution for building a smart supply chain (Barykin et al., 2020; 2021; AlMulhim, 2021; Busse et al., 2021; Ivanov and Dolgui, 2021). The digital twin (DT) concept uses advanced digitalization to mirror the physical world in the virtual world and has been named one of Gartner's Top 10 Strategic Technology Trends from 2017 to 2019 (Panetta, 2017; 2018; 2019). Gartner predicts that half of the world-famous industrial companies will implement DTs by 2021, thereby improving the companies' effectiveness by 10% (Pettey, 2017). According to Thomas Kaiser, SAP Senior Vice President of the Internet of Things (IoT), "DTs are becoming a business imperative, covering the entire lifecycle of an asset or process and forming the foundation for connected products and services; companies that fail to respond will be left behind" (Marr, 2017).

The DT concept was developed to enable smart manufacturing (Glaessgen and Stargel, 2012; Shafto et al., 2012; Grieves, 2015), but the DT's ability to mirror, predict, and optimize complex systems allows it to be effective even beyond manufacturing systems. As explained in Section 3, the DT concept and the smart supply chain perfectly complement each other. Therefore, building a DTSC is the solution to realize the smart supply chain. In a DTSC, the virtual supply chain and the physical supply chain are entangled (Rehana, 2018). All stakeholders can access the real-time status of physical entities (e.g., stocks, procurement, and sales). Managers can simulate and implement decisions in the virtual world before executing them in the physical supply chain. The primary objective is to enable the supply chain to operate with better performance and higher efficiency.

This article contributes to the literature by investigating the following questions:

(1) What is the DT concept and how is it being used and exploited in supply chain management (SCM)? Section 2 discusses these topics.

(2) What are the basic components to build a DTSC? Why do these components satisfy the requirements of the smart supply chain? Section 3 discusses these topics.

(3) How can the existing SCM theories be applied, and what are the new research opportunities in the DT context? Section 4 presents these topics.

(4) What are the implications and future challenges when implementing the DTSC in actual business environments? These matters are analyzed in Section 5 by presenting a motivating case of JD.COM.

## 2 Literature review

In this section, we first review the concept development of the DT and the properties of an efficient DT. Then, we proceed to review the current DT applications in SCM.

### 2.1 History of the DT concept

The DT concept has been developed by practitioners and researchers after 20 years of effort. Table 1 summarizes the conceptual development from 2002 to 2020. Two notable trends have reshaped the understanding of a DT. The first trend is a shift from viewing the DT concept as a single methodology to a multidisciplinary integration. The key driver behind this change was the gradual broadening of the scope of the DT application. The original concept was based on product lifecycle management (PLM) in smart manufacturing and aerospace engineering (Grieves, 2005; 2006; 2011; Glaessgen and Stargel, 2012; Shafto et al., 2012). As the application scope widened, the specific application domain lost emphasis. Researchers and practitioners proposed general concepts, such as "virtual information constructs" (Grieves and Vickers, 2017) and "the linked collection of the relevant digital artifacts" (Boschert and Rosen, 2016). Therefore, simulation alone is incapable of building all the required virtual models and incorporating multidisciplinary approaches as needed. The paradigm's organic connection to new and emerging technology enables the DT concept to play a pivotal role in developing intelligent enterprise and supply chains (Anasoft, 2019).

The second trend involved a shift in the focus of the DT concept from being "tech-oriented" to "decision-oriented". Although the DT concept originally focused on virtual modeling (Grieves, 2015; Boschert and Rosen, 2016; Grieves and Vickers, 2017), new approaches have emphasized the paradigm's capability to provide optimal decisions and prospective insight that is data-driven (Stanford-Clark et al., 2019; Stark and Damerau, 2019; Olcott and Mullen, 2020). As the Acatech Industrie 4.0 Maturity Index (Schuh et al., 2017) suggested, predictive capacity and adaptability — including automated decision-making and actions — are based on connectivity and visibility that result from creating connected virtual counterparts.

We summarize the properties of a DT on the basis of these principles. First, the core property of an efficient DT is to establish digital representations of physical entities and processes at different scopes (Boschert and Rosen, 2016; Stark and Damerau, 2019; Qi et al., 2021). A DT overcomes data silo issues in traditional enterprise

**Table 1** History of the digital twin (DT) concept

| Year | Event |
|---|---|
| 2002 | Conceptual ideal for product lifecycle management (PLM) (a presentation by Dr. Grieves) |
| 2005 | Mirrored Spaces Model (Grieves, 2005) |
| 2006 | Information Mirroring Model (Grieves, 2006) |
| 2011 | Digital twin (Grieves, 2011) |
| 2012 | A DT is an integrated multiphysics, multiscale, probabilistic simulation of an as-built vehicle or system that uses the best available physical models, sensor updates, and fleet history to mirror the life of its corresponding flying twin. The DT is ultra-realistic and may consider one or more important and interdependent vehicle systems, including airframe, propulsion and energy storage, life support, avionics, and thermal protection. The concept is proposed by NASA and the US Air Force (Glaessgen and Stargel, 2012; Shafto et al., 2012) |
| 2015 | A virtual representation of what has been produced (Grieves, 2015) |
| 2016 | A set of virtual information constructs (Grieves and Vickers, 2017) (first online in 2016) |
| 2016 | The linked collection of the relevant digital artifacts including engineering data, operation data, and behavior descriptions via several simulation models (Boschert and Rosen, 2016) |
| 2019 | A DT is a digital representation of an active unique product (real device, object, machine, service, or intangible asset) or unique product–service system (a system consisting of a product and a related service) that is composed of selected characteristics, properties, conditions, and behaviors by means of models, information, and data within a single or even across multiple life cycle phases. The concept is proposed by the International Academy for Production Engineering Encyclopedia of Production Engineering (Stark and Damerau, 2019) |
| 2019 | A DT is a dynamic virtual representation of a physical object or system, usually across multiple stages of its lifecycle. It uses real-world data, simulation, or machine learning models, combined with data analysis, to enable understanding, learning, and reasoning. DTs can be used to answer what-if questions and present insights in an intuitive way. The concept is proposed by IBM (Stanford-Clark et al., 2019) |
| 2020 | A DT is a virtual representation of real-world entities and processes, synchronized at a specified frequency and fidelity. The concept is proposed by the Digital Twin Consortium (Stanford-Clark et al., 2019) |

management systems and provides a holistic understanding (Olcott and Mullen, 2020). Second, the availability of real-time data enables high-frequency synchronization between physical entities and processes and digital representation. A DT can leverage historical and real-time data to simulate the system’s past, present, and future behavior. Finally, armed with predictive analysis and optimization tools, a DT can provide useful information and knowledge, allowing intelligent responses to sudden and unexpected situations.

### 2.2 State-of-the-art DTSC

We search publications on the Web of Science database with search terms including “digital twin(s)” and “supply chain” from January 2016 to October 2021. Retrieved articles have discussed the following issues: (1) What is a DTSC? (2) What are the advantages that the DT concept brings to SCM? And (3) is there any case to illustrate the value of implementing a DTSC?

**What is a DTSC?** The concept of DTSC has been considered by several articles. According to Busse et al. (2021), a DTSC is “a digital simulation model of a real logistics system, which features a long-term, bidirectional and timely data-link to that system”. The authors propose a general framework, including modules of supply chain, optimization, simulation, reporting, and interface. Ivanov and Dolgui (2021) identify a DTSC as “a computerized model that represents network states for any given moment in real time”. Haag and Simon (2019) suggest that implementing DTs provides the possibility for companies to model real-world assets and their interactions in arbitrary magnitude and level of detail. Li et al. (2020) propose a five-dimensional framework of a sustainable business model under the DTSC concept. Recently, Kalaboukas et al. (2021) propose the concept of cognitive DTs for SCM, which are capable of predicting trends and flexible enough in dynamic environments. In addition to these general conceptual development, the DTSC concept has been discussed in several specified application fields, including circular supply chain (Preut et al., 2021), food supply chain (Smetana et al., 2021; Shoji et al., 2022), global port management (Wang et al., 2021), and logistics (Lee and Lee, 2021; Moshood et al., 2021; Park et al., 2021). In summary, the core functionality of a DTSC is to provide an integrated and holistic view, which enables all stakeholders to contribute jointly to the creation across different supply chain stages (Ducree et al., 2020; Yang et al., 2020).

**What are the advantages that the DT concept brings to SCM?** A number of articles suppose that the DT strategy is expected to lead the digital transformation of the supply chain to the next stage (Cozmiuc and Petrisor, 2018; Ghobakhloo, 2018; Reeves and Maple, 2019; Marmolejo-Saucedo et al., 2019; Beltrami et al., 2021; Kenett and Bortman, 2021). The first advantage that the DT concept brings to SCM is improved connectivity. Sharma et al. (2021) suppose that the DT concept connects different life stages in the chain. The authors review strategies to balance efficiency and cost to enable such connectivity. According to Pehlken and Baumann (2020), DTs help provide production information to a variety of stakeholders in the recycling industry and therefore increase the impact on sustainability. Zafarzadeh et al. (2021) focus on production logistics. The authors analyze 142 articles and conclude that the application of the DT concept that provides a connected and holistic view of production logistics is important. To realize connectivity successfully, a DTSC needs a unified data management approach to create a new integrated vision of the supply chain (Avventuroso et al., 2017; Landolfi et al., 2017; Gupta et al., 2020; Autiosalo et al., 2021).

The second advantage that DTs bring to SCM is improved end-to-end visibility (Moshood et al., 2021). As mentioned by Hegedus et al. (2019), a DTSC can serve as a retrofittable and cost-effective solution for asset tracking across supply chain, which is a highly desirable functionality in SCM. Wang et al. (2020) conceptualize the idea of DTSC for overcoming the limited visibility of the logistics process in non-high-tech industries. Tozanlı et al. (2020) study how the DT concept facilitates a data-driven trade-in pricing policy in fully transparent platforms. Wang et al. (2021) present information visualization for using DTs in global port management. Chen and Huang (2021) identify information asymmetry as the main challenge that restricts the development of closed-loop supply chain. The authors analyze 288 articles and find that the DT concept is conducive to solving problems with information asymmetry in remanufacturing supply chains. In particular, blockchain technology for SCM in the DT context can be used for security and privacy (Kanak et al., 2019; 2020; Greif et al., 2020; Joannou et al., 2020; Leng et al., 2020; Deng et al., 2021; Ho et al., 2021).

The third advantage is DTs' capability of increasing supply chain agility and resilience. Several researchers have discussed the concept of DTSC in managing disruption risks (Ivanov and Dolgui, 2019; 2021; Ivanov et al., 2019). Seif et al. (2019) note that the DT concept enables agility because a modular way of modeling is applied. Golan et al. (2021) address gaps in modeling supply chain resilience in general and specifically for vaccine production. The authors suggest that adopting a DTSC helps supply chain managers to quantify tradeoffs between efficiency and resilience under disruption better. Barykin et al. (2020) review the optimization and simulation methods that are used to evaluate the impact of potential failure on supply chain performance in the DTSC context.

Considering that supply chains are becoming increasingly complex, several researchers discuss how to deal with the complexity and enable intelligent supply chain in the DT context. Simulation tools are useful to represent the interdependencies in complex supply chains in the DT context, including discrete event simulation (D'Angelo and Chong, 2018; Dobler et al., 2020; Dutta et al., 2021; Pilati et al., 2021; Wilson et al., 2021), agent-based simulation (Gorodetsky et al., 2019; Clark et al., 2020; Orozco-Romero et al., 2020), and hybrid simulation models (Makarov et al., 2021). Frazzon et al. (2020) address distributed decision-making under a socio–cyber–physical system perspective in the DTSC context. Shen et al. (2020) discuss how the DT concept supports collaborative intelligent manufacturing and the SCM, including resilient collaborative supply network design, collaborative planning of production and distribution, dynamic reconfiguration and rescheduling, and remote maintenance. Baruffaldi et al. (2019) exploit optimization and simulation techniques to quantify how information availability impacts warehousing operations, which results in the creation of a DT of the warehouse. Cavalcante et al. (2019) combine machine learning and simulation to create a DTSC for improving sourcing decisions. As mentioned by Barykin et al. (2020), no single approach builds the concept of a DTSC, and hybrid and data-driven decision-making algorithms are identified as a key enabler to realize the value of the DT concept in SCM (de Paula Ferreira et al., 2020; Andronie et al., 2021).

**Is there any case to illustrate the value of implementing a DTSC?** The value of DTSC implementation has been validated conceptually and tested in several experimental environments in a variety of application domains. A number of articles have investigated how the DTSC concept improves supply chain agility under COVID-19 pandemic (Ivanov, 2020; Burgos and Ivanov, 2021; Ivanov and Dolgui, 2021; Nasir et al., 2021). In particular, creating a DTSC helps policymakers deal with situations in the COVID-19 pandemic (Nasir et al., 2021). Another application domain is the food supply chain, in particular, fresh food delivery (Defraeye et al., 2019; 2021; Onwude et al., 2020). With the help of a DTSC, producers, retailers, and consumers can continuously monitor and control quality evolution during fresh food postharvest life. The DTSC concept has also been applied in the literature on pharmaceutical (Marmolejo-Saucedo, 2020; Santos et al., 2020), semiconductor (Ehm et al., 2019; Moder et al., 2020a; 2020b), aircraft manufacturing supply chain (Mandolla et al., 2019; Heim et al., 2020), the coal industry (Semenov et al., 2020), and tobacco industry (Shen et al., 2021).

We identify the gaps in the literature and try to address the following issues in this article. First, in spite of the advanced conceptual development of the DTSC, questions about the actual detailed architecture of a DTSC have to be studied (Busse et al., 2021). We discuss the basic components to build a DTSC in Section 3. Second, the existing literature has discussed how to use mature methods to enable intelligent decision-making in a DTSC (e.g., simulation and machine learning), whereas we extend the discussion by showing how existing SCM theories can be applied. We also discuss new research opportunities in the DT context in Section 4. Third, the proposed frameworks are validated either conceptually or in experimental environments. We illustrate how the DTSC concept helps in large-scale real-life business environment in Section 5.

## 3 Building a DTSC

In this section, we describe how to build a DTSC. To begin, we briefly review the definition of supply chain and SCM. Then, we explain the structure of a DT and identify the corresponding components in a DTSC. Finally, we discuss how considering these components satisfies the properties of the smart supply chain.

According to Christopher (2011), a supply chain is “a network of organizations that are involved, through upstream and downstream linkages, in the different processes and activities that produce value in the form of products and services in the hands of the ultimate customer”. Generally, a supply chain consists of suppliers, manufacturers, distributors, retailers, and customers. Products or services flow downstream to end customers. The flow of information and funds is bidirectional between supply chain nodes. Therefore, the purpose of SCM is to manage the flow of products, information, and finances associated with a series of inter- and intra-enterprise activities. Such activities typically include procurement planning, manufacturing strategy, customer order management, inventory management, logistics management, and marketing (Chandra and Kumar, 2000; Min and Mentzer 2000; Lummus et al., 2001).

Considering the properties of an efficient DT and the definitions of supply chain and SCM, stakeholders must identify (1) the scope of physical entities that should be the focus of the supply chain; (2) the fidelity applied to understand the current status of these physical entities in virtual space; and (3) the key sources of data used to synchronize the physical supply chain and the virtual one. Once these information are defined, a DTSC prototype may be built.

In a DTSC, we focus on two types of physical entities. First, a DTSC must include the supply chain nodes, which are enterprises involved in the supply chain. Given that the aim of a DTSC is to enhance the performance and competitiveness of the entire chain, the physical entities must include all the enterprises, beginning with suppliers and ending with customers. Second, products, properties, and other valuable items should be considered. Products are the core output that brings profit and competitiveness to the chain, and it is an indispensable part of the physical entities in a DTSC. Properties are the items needed to produce and deliver products, and they belong to enterprises in the supply chain. For example, a manufacturing company owns the raw materials and equipment needed to produce its goods. Other valuable items are facilities that add value to the business but belong to external organizations, such as the trucks of a third-party logistics company. By including these two types of physical entities, we can mirror the status, the intra-enterprise operations, and the inter-enterprise communications of a firm, and therefore, mirror the entire business operation in virtual space.

Having clarified the two types of physical entities considered in a DTSC, we must discuss the fidelity that should be used to analyze these physical entities in virtual space. We suggest creating a virtual or digital supply chain that reflects the physical supply chain in the following two aspects: (1) the basic properties of the physical entities and (2) the inter- and intra-enterprise business processes. The basic properties are the static attributes of the physical entity, such as organizational structure, strategic objectives, and location. For the product, we consider attributes such as appearance, size, and functions. For manufacturing equipment, the relevant properties usually include function and power consumption. As an example, for a truck used in transportation, endurance and capacity are taken into consideration.

The key steps in mirroring the physical supply chain in virtual space is to define the inter- and intra-enterprise business processes, such as procurement, production, distribution, and marketing. To define a business process clearly, we suggest asking the following questions:

- What are the goals of the business process?
- Is it an intra-enterprise business or an inter-enterprise business?
- What is the standard procedure of the business activity?
- At each step, which enterprise or department makes decisions or takes action?
- What policies, regulations, and limitations must be considered before decisions are made or actions are taken?
- How should the performance of the business process be evaluated?

If both basic properties and business processes are properly defined, then we can develop a virtual supply chain that exactly mirrors the structure and functions of the physical supply chain.

The synchronization refers to the transmission of bidirectional information. Generally, the data used in the physical supply chain fall into one of the following two categories: (1) static data related to the basic properties of physical entities or (2) real-time, dynamic data generated in business operations. Static data are used to ensure that the virtual supply chain shares the same structure and properties as the physical supply chain. Real-time, dynamic data are used to synchronize the status and processes in the virtual world. For example, the location of a truck and traffic conditions are updated in real time; hence, the estimated arrival time is updated continuously and is precise.

Real-time data are collected not only by sensors and other IoT equipment, which is typical with manufacturing systems, but also by online systems, such as procurement management systems and order management systems. In the virtual world, data are simulated. On the basis of the analysis of the actual data in the physical supply chain and the simulated data in the virtual supply chain, we obtain results that provide valuable information and knowledge. Information and knowledge are then transferred to the physical world to support intelligent decision-making and the efficient implementation of decisions.

A DTSC is proposed as a solution to the smart supply chain, because the features of a DT and the requirements of the smart supply chain match each other. In a DTSC, the physical supply chain is connected by smart sensors or online systems and collects specific data and information that enable the virtual supply chain to mirror the static properties and dynamic business processes of the physical supply chain. Connectivity is therefore achieved. As the most important features in the DT, real-time data acquisition and implementation allow the connection between the physical and virtual supply chain to synchronize operation dynamics, which increases the supply chain visibility. The synchronized data provide opportunities to monitor, analyze, control, and optimize the supply chain and results in up-to-date virtual simulation and optimization that provide agility. A DTSC actively operates in the entire business process throughout the supply chain. Therefore, a DTSC jointly optimizes the supply chain across different stages and establishes an integrated supply chain. Predictive analytics are “the capabilities used to model and simulate current and future conditions, considering operating conditions to test future-state scenarios” (Klappich, 2019). Consequently, predictive analytics of a DTSC allow decision-makers to look forward instead of backward and allow the supply chain to be intelligent.

## 4 Digital twin-driven smart supply chain: New research opportunities

Building on the explanation of a DTSC, we use this section to discuss new research opportunities to implement a smart DTSC. In Section 4.1, we focus on supply chain modeling, including demand, supply, and risk modeling. In Section 4.2, we center our discussion on making real-time decisions in SCM, and in Section 4.3, we explain data usage in supply chain collaboration.

### 4.1 Supply chain modeling

A DTSC enables an accurate and timely representation of the supply chain status. Considering analytical tractability and that data may be limited, partial, or difficult to access, simplified assumptions about demand, supply, and risks are used in supply chain modeling rather than an accurate characterization of reality. With the DTSC’s capability to mirror dynamics, assumptions that were commonly and previously used need to be reviewed, and a structural and theoretical analysis is required.

First, to take full advantage of real-time demand signals and supply conditions in a DTSC, novel methods that model supply and demand in a nonlinear way are required. For example, to model random supply and demand, Feng and Shanthikumar (2018) propose a new approach that transforms nonlinear supply and demand functions into linear functions on a higher dimension. Data-driven optimization is another research tool that addresses the complex supply and demand data by solving mathematical programming problems directly using observed data (Bertsimas and Thiele, 2006). Without assuming a particular distribution, one-step optimization is used with the collected data rather than the traditional two-stage approach of optimization after estimation (Liyanage and Shanthikumar, 2005).

Levi et al. (2015) illustrate an application of this idea in modeling demand with unknown distribution. The authors study the newsvendor problem in which the only information is a random sample of demand. The accuracy of the proposed sample average approximation approach is ensured by a tight bound. Compared with demand signals, more research is needed to understand the modeling capacity from data-driven approaches. Currently, most studies assume the infinite capacity according to de Kok et al. (2018). Feng and Shanthikumar (2018) consider capacitated supply chains and concluded that three ways can be used to model capacity: (1) deterministic supply, (2) all-or-nothing supply, and (3) proportional random yield.

Second, building a DTSC can innovate the approach to risk modeling. Generally, discrete events are used to model risks, and risks are assumed to propagate between adjacent nodes in supply networks. Garvey et al. (2015) apply Bayesian networks to measure risk propagation in a supply network. These measures can be combined with other problems (e.g., inventory management and network design) to develop globally optimal solutions. The authors focus on binary risk and assume that all conditional probability distributions are well-defined. In a DTSC context, the propagation of risk signal (information) and the actual risk (event) can be asynchronous. The risk signal can be synchronized instantaneously throughout the entire supply chain, but the actual influence of the risk needs time to propagate. New modeling approaches are required for companies to understand asynchronous signal and event. Analytics need to be developed to predict when actual risk happens to allow companies to prepare.

Third, model adaptability with real-time data requires future research. Supply chains today are facing a changing environment. Simulation models in a DTSC are a promising way to manage the complexity and uncertainty of reality (Tohamy, 2019). According to Rajagopal et al. (2017), simulation methods that consider disruption and supply risks in a multi-period setting are applied in supply chain network design, risk propagation analysis (Wu et al.,

2013; Bueno-Solano and Cedillo-Campos, 2014; Chen et al., 2015), facility location, and inventory management (Colicchia et al., 2010; Schmitt and Singh, 2012; Sarkar and Kumar, 2015). Despite these advances, we still require novel approaches to model adaptability, that is, the ability to learn continuously from real-time status and to update models constantly.

Currently, data-driven approaches have been widely adopted to enable demand learning. To name a few, Deng et al. (2014) use the Bayesian method in statistically learning service-dependent demand. In Cao et al. (2019), customers' arrival rate (demand) is learned in a Bayesian method. Harrison et al. (2012), Ghate (2015), and Chen et al. (2017) propose other Bayesian demand learning methods. Ma et al. (2020) propose a framework of data-driven sustainable smart manufacturing based on demand response for energy-intensive industries. Pereira and Frazzon (2021) propose a data-driven approach that combines machine-learning demand forecasting and operational planning simulation-based optimization to synchronize demand and supply adaptively in omni-channel retail supply chains. In spite of the advanced data-driven methods, leveraging real-time data to update simulation models in a changing environment remains an unresolved problem (Hong and Jiang, 2019). Moreover, little has been done to identify how to leverage demand and supply information through product lifecycle rather than a single demand/supply data point (Ma et al., 2020).

In summary, to take full advantage of real-time data and information in a DTSC, we need novel methods to capture supply, demand, and risks accurately in supply chain modeling. To manage the changing environment, increasing model adaptability remains an important issue for future research.

### 4.2 Real-time supply chain optimization

In a DTSC, the physical status and the digital model are frequently synchronized, which requires real-time and system-level instantaneous optimization of available information (Olsen and Tomlin, 2020). Using offline results directly is a promising way to respond to environmental changes under tight computation budgets. Lowrey et al. (2018), Hong and Jiang (2019), and Jiang et al. (2020) explain this idea. A simple illustration of this principle in inventory management is the use of radio-frequency identification technology to collect real-time data and inventory policies predefined at the retail store (Bottani et al., 2017). The real-time data are used to track product traceability, product history, amount of sales, product availability, and inventory. With the inventory and product availability information, a store can implement inventory policies with continuous review as soon as an inventory status change is detected in real time.

Making all decisions in real time is not necessary, and choosing the right synchronization rate is required. In practice, "real-time" means that the time between real-world changes is negligible with respect to the need and intended usage of the digital models by applications or users (Minerva et al., 2020). Generally, two strategies are applied to trigger the algorithms in real-time problems: Time trigger strategy (e.g., making decisions every minute) and event trigger strategy (e.g., making decisions when a new demand arrives). Heemels et al. (2012) conduct a debate on time trigger (periodic) and event trigger in system control. Despite the computation delays explicitly considered by the authors, more types of delay occur in practice. Figure 1 shows a basic decision scheme in a DTSC. According to Power (2011), "real time" in practice always has latency between (1) physical supply chain changes, (2) the reflection of physical supply chain change in data in one or more systems of record (the availability of real-time data), and (3) the availability of changed data to current optimization models. The above summary considers only the process from the real world to virtual representations. If the feedback process is taken into consideration, additional latency occurs between (4) the availability of decisions proposed by the optimization

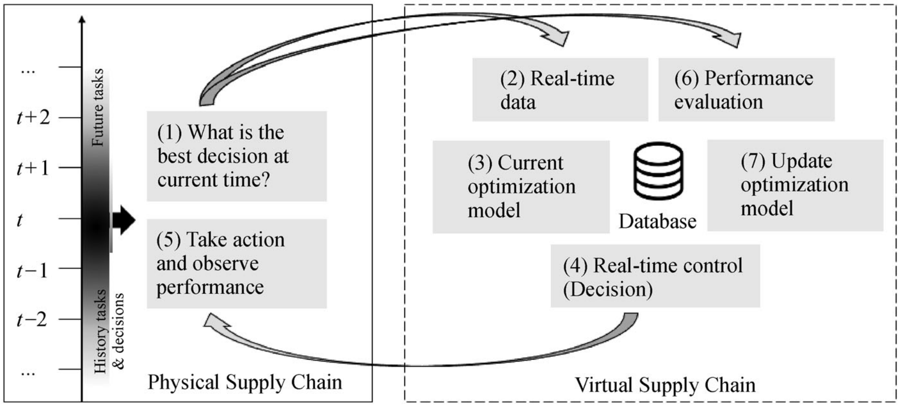

**Fig. 1** Basic real-time decision scheme in a DTSC.

model and (5) the implementation of the decision by an actuator or an operator and the actual changes.

In a DTSC context, the length of the decision period varies among different applications. For example, Guo et al. (2017) focus on the global network configuration problem and provide an online-learning approach of joint optimization to change a labor-intensive and error-prone configuration to one that is optimally designed. Ulmer (2019) reoptimizes vehicle routing as a reaction to new customer requests and in anticipation of future requests. Sung et al. (2021) apply two different offline path planning algorithms to generate different training path data sets and to improve the online path planner performance. In some contexts, the length of a decision period may be short. For instance, automated guided vehicles are controlled at the millisecond level, and the reconfiguration of a production line to respond to urgent orders must be determined in light of the takt time. An open question is how to combine different trigger strategies, considering the above latency, to achieve timely synchronization and response in a practical context. Moreover, closing the loop between offline models and online feedback has unanswered questions. For example, considering the time to (6) validate performance and (7) update optimization models, how often should offline models be checked and updated? The discussion on model adaptability in the previous section is closely related to this question.

### 4.3 Data usage in supply chain collaboration

We discuss data-driven modeling and decisions in previous sections. In this section, we focus on the proper data usage in supply chain collaboration. According to the *Accenture Technology Vision* report (Daugherty et al., 2021), 87% of executives identify that the value of a DTSC is in their organization's increased ability to reflect information about their supply chains, instead of their own organizations. Inevitably, data usage issues in a DTSC must be addressed.

Questions related to data ownership and privacy remain unanswered. When a company builds a DTSC using a DT vendor and allows their supply chain partners to upload data to the platform, ownership (i.e., vendors, organizations, or both) of this industrial data is unclear (Internet of Business, 2017). Companies face the risk of inadvertently signing imbalanced agreements with DT vendors. Furthermore, companies must carefully manage sensitive customer information, which is increasingly exposed to criminal threats (Fuller et al., 2020) and regulated by laws. For example, the *European Union's General Data Protection Regulation* regulates personal data privacy and security. In particular, these new regulations require data controllers (companies) to explain their data use to data subjects (customers) (European Union, 2018). China's government also has proposed new data security laws to regulate Internet information services and algorithmic recommendations (Wang, 2021).

In addition, supply chain collaboration with real-time information and the use of new technologies require more research. The existing literature considers the communication of demand and inventory data, but other types of data have not been fully analyzed. A DTSC synchronizes not only demand and inventory data, but also dynamic behaviors, including manufacturing, distribution, marketing, and logistics. Should the effect of this data sharing on the behaviors and costs be studied as well? Should real-time data be shared in the form of statistical information or the original data? How often should real-time information be communicated? How does the automated consensus mechanism provided by blockchain affect the coordination behaviors in the supply chain? These questions need to be answered to improve the application of DTSC.

## 5 Case introduction

In this section, we provide a motivating case study from JD.COM, China's largest retailer by revenue, in reconfiguring the supply chain network during the COVID-19 pandemic using a DTSC platform. As a result, applying a DTSC platform significantly improves the response efficiency of JD.COM.

### 5.1 Background

Nowadays, different emerging trends, such as retail decentralization, community group buying, social shopping, and live commerce, are accelerating the interaction and integration of retail and manufacturing. As a result, the supply chain structure is becoming highly complicated. JD.COM operates 41 mega "Asia No. 1" logistics parks in China, with nearly 1300 warehouses and over 9 million self-operated stock keeping units (SKUs). The scope of products covers consumer packaging goods, information appliances, home appliances, clothing, fresh food, books, and automobiles. The transportation network includes multiple transportation methods, containing land transportation and shipping. JD.COM extends the supply chain planning and operation to both upstream and downstream, by using digital and intelligent technologies and multichannel models. To manage and optimize the operations, traditional supply chain planning methods and algorithms are facing increasing challenges.

The challenge was amplified by the COVID-19 outbreak from the end of 2019. First, the pandemic caused exceptional demand for masks, alcohol, household cleaning products, and food. Second, transportation networks were interrupted due to lockdowns. Third, products were out of stock because of the shortage of labor and raw materials and logistical disruptions. To address these problems, JD.COM applied a DTSC platform to support its business. According to Curtis Liu, Vice President of

JD.COM and President of JD Intelligent Supply Chain, crises such as epidemics have highlighted the importance of a smart supply chain. Through the applications of a DTSC, data-driven models combined with simulation facilitate the quick evaluation and adjustment of supply chain planning strategies. Building a DTSC will become an important trend for managing future supply chains.

### 5.2 The DTSC platform in JD.COM

JD.COM's DTSC platform builds end-to-end digital representations for the entire supply chain. Compared with traditional retail supply chains, which consist of several distinct stages, JD.COM has an integrated supply chain structure, where products are directly delivered from factories and manufacturers to consumers through a single jd.com platform. This integrated supply chain structure has a higher level of collaboration, more effective information sharing, and a higher level of agility than existing supply chain structure (Shen and Sun, 2021). The DTSC platform is well established to support this integrated supply chain structure by effectively connecting internal systems in JD.COM and external systems to offer a holistic value throughout the supply chain, as shown in Fig. 2. The data sources used to synchronize the physical supply chain and the digital models include network configuration platform, procurement system, transfer system, and fulfillment system. Optimization algorithms and simulation algorithms work together to support integrated supply chain design and intelligent operations. By directly connecting with decision and execution systems, these optimized insights can be quickly implemented to respond to changes. Meanwhile, insights are visualized for better understanding. The DTSC platform is expected to support a wide range of business decision-makings in JD.COM, from long-term strategies, to mid-term plans and short-term operations. The next subsection takes the supply chain network reconfiguration as an example.

### 5.3 The DTSC platform for enabling supply chain network reconfiguration

In this section, we illustrate how the DTSC platform improves operational efficiency to address the challenges during the COVID-19 pandemic. In a typical two-level supply chain network, upstream distribution centers might suddenly be unable to distribute goods to warehouses because regional outbreaks resulted in travel restrictions. In such cases, JD.COM had to reconfigure its supply chain network in response to the disruptions. Orders could be replenished by other alternative distribution centers or the backorder strategy could be used. Adopting alternative distribution centers negatively affected the order fulfillment rate in its original service area and lead to significant additional transportation costs. Considering the unpredictable outbreaks and varying sales and inventory situations of massive SKUs in different regions, whether and how to reconfigure the supply chain network for cross-regional distribution were difficult to assess. The DTSC platform is capable of comprehensively and deeply considering conflicting objectives and quickly simulating and optimizing different strategies. Using the DTSC platform entails the following steps:

- Internalizing simulation models in the DTSC platform. The JD.COM DTSC platform supports the creation of models from the physical level, time level, process level, and cost level to map the real environment as accurately as possible. The platform uses significant data technology to

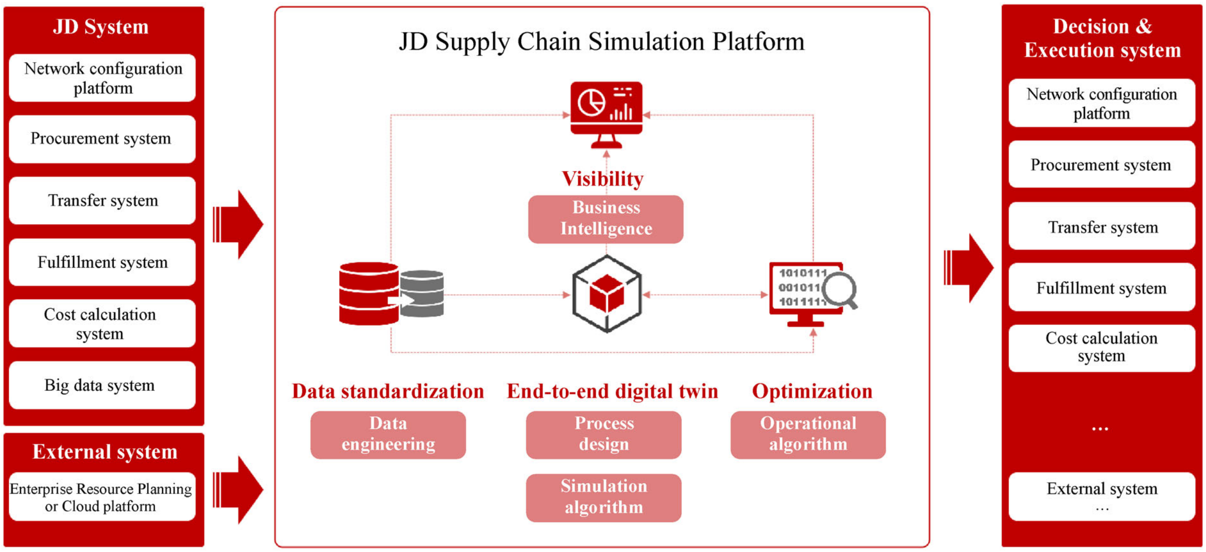

**Fig. 2** Framework of JD.COM's DTSC platform.

create the basic data automatically for simulation models from the production system. The basic information includes the scope of products, the scope of the region, the distribution center and warehouse information, and the customer type.

• Mirroring the current business. After initialization, simulation models require detailed structure and parameters to mirror the real system accurately. Additional settings include the current network structure and replenishment strategy. Taking one of the cases during the COVID-19 pandemic as example, the demand in Shijiazhuang area was replenished by two distribution centers in Shijiazhuang and Beijing. By carefully calibrating the parameters, the simulation models in the platform were able to capture the physical supply chain in terms of some key indicators (e.g., local order fulfillment rate and transportation costs) with an average accuracy of 96%.

• Setting candidate plans. Taking the same example, owing to travel restrictions in Beijing, using the distribution centers in Tianjin was set as the alternative plan.

• Analyzing and visualizing the results. JD.COM applied advanced algorithms to improve the delivery network design (Kang et al., 2021). In the above case, the alternative solution was evaluated by the inventory turnover rate, inventory availability, order split rate, local order fulfillment rate, ratio of same day delivery, and total costs. As a result, only part of the SKUs was replenished by the distribution center in Tianjin to remain at a high service level.

In summary, JD.COM's DTSC platform significantly improves operational efficiency in response to sudden disruptions in the supply chain during the COVID-19 pandemic. The platform supports integrated supply chain planning instead of optimizing the single supply chain stage independently. The platform achieves a breakthrough in the end-to-end supply chain representation in the virtual space. Facilitated by data-driven optimization tools, the response time to disruptions is shortened by an average of 50%. Previously, the evaluation of supply chain reconfiguration with nearly 200000 SKUs took several days. The running time is reduced to less than an hour with the help of big data analytics and optimization tools in the DTSC platform.

### 5.4 Discussion

Encouraged by the successful application in the supply chain network reconfiguration during the COVID-19 pandemic, JD.COM is enthusiastic about applying the DTSC platform on a large scale to realize the smart supply chain. First, the DTSC platform is developed to model JD.COM's nationwide supply chain network, which requires large-scale simulation and optimization tools. Second, the platform is expected to realize high-frequency synchronization between physical supply chain and digital models. The daily operational efficiency can be improved by exploring real-time data. Third, analytics will be used proactively to foresee potential disruptions and to get JD.COM fully prepared.

## 6 Conclusions

Given the innovative DT concept, the opportunities to build and improve the smart supply chain are considerable. Practitioners should embrace the innovative DT technology to build a DTSC from the ground up. Realizing the smart supply chain vision takes time, but the transformation is worthwhile. For future study, researchers are encouraged to solve new problems presented by the smart supply chain. We need new modeling approaches and decision-making patterns to make full use of real-time data and adapt to the changing environment. We must explore using a DTSC to transform data and information into knowledge and value. To reduce risks and increase agility, new problems related to predictive analytics for the smart supply chain must be addressed. Our discussion is not exhaustive. We expect more practical and academic focus on the DT concept in order to fully realize the benefits of the DTSC.

**Open Access** This article is licensed under a Creative Commons Attribution 4.0 International License, which permits use, sharing, adaptation, distribution and reproduction in any medium or format, as long as you give appropriate credit to the original author(s) and the source, provide a link to the Creative Commons licence, and indicate if changes were made.

The images or other third party material in this article are included in the article's Creative Commons licence, unless indicated otherwise in a credit line to the material. If material is not included in the article's Creative Commons licence and your intended use is not permitted by statutory regulation or exceeds the permitted use, you will need to obtain permission directly from the copyright holder. To view a copy of this licence, visit http://creativecommons.org/licenses/by/4.0/.
